# No association of a risk variant for severe COVID-19 with HIV protection in three cohorts of highly exposed individuals

**DOI:** 10.1093/pnasnexus/pgac138

**Published:** 2022-08-04

**Authors:** Manuela Sironi, Rachele Cagliani, Mara Biasin, Sergio Lo Caputo, Irma Saulle, Diego Forni, Luis Miguel Real, Juan Antonio Pineda, Almudena Exposito, María Eugenia Saez, Faruk Sinangil, Donald Forthal, Antonio Caruz, Mario Clerici

**Affiliations:** Scientific Institute IRCCS E. MEDEA, Bioinformatics, Bosisio Parini, 23842 Lecco, Italy; Scientific Institute IRCCS E. MEDEA, Bioinformatics, Bosisio Parini, 23842 Lecco, Italy; Laboratory of Immunobiology, Department of Biomedical and Clinical Sciences L. Sacco, University of Milan, 20157 Milan, Italy; Unit of Infectious Diseases, Department of Clinical and Experimental Medicine, University of Foggia, 71122 Foggia, Italy; Laboratory of Immunobiology, Department of Biomedical and Clinical Sciences L. Sacco, University of Milan, 20157 Milan, Italy; Scientific Institute IRCCS E. MEDEA, Bioinformatics, Bosisio Parini, 23842 Lecco, Italy; Unidad de Enfermedades Infecciosas y Microbiología Clínica. Hospital Universitario de Valme, 41014 Sevilla, Spain; Departamento de Especialidades Quirúrgicas, Bioquímica e Inmunología. Facultad de Medicina. Universidad de Málaga, 29010 Málaga, Spain; Centro de Investigación Biomédica en Red de Enfermedades Infecciosas (CIBERINFEC), 28029 Madrid, Spain; Unidad de Enfermedades Infecciosas y Microbiología Clínica. Hospital Universitario de Valme, 41014 Sevilla, Spain; Centro de Investigación Biomédica en Red de Enfermedades Infecciosas (CIBERINFEC), 28029 Madrid, Spain; Departamento de Medicina. Facultad de Medicina. Universidad de Sevilla. Instituto de Biomedicina de Sevilla (IBiS), 41013 Sevilla, Spain; Unidad de Inmunogenética, Genética, Departamento de Biología Experimental, Universidad de Jaén, 23071 Jaén, Spain; Centro Andaluz de Estudios Bioinformáticos, 41092 Sevilla, Spain; Global Solutions for Infectious Diseases, Lafayette, 94549 CA, USA; Division of Infectious Diseases, Department of Medicine, University of California, Irvine School of Medicine, Irvine, 92697 CA, USA; Unidad de Inmunogenética, Genética, Departamento de Biología Experimental, Universidad de Jaén, 23071 Jaén, Spain; Department of Physiopathology and Transplantation, University of Milan, 20122 Milan, Italy; Don C. Gnocchi Foundation ONLUS, IRCCS, 20133 Milan, Italy

**Keywords:** HIV, COVID-19, genetic risk factor, exposed seronegative individuals

## Abstract

An extended haplotype on chromosome 3 is the major genetic risk factor for severe COVID-19. The risk haplotype, which was inherited from Neanderthals, decreases the expression of several cytokine receptors, including CCR5. Recently, a study based on three general population cohorts indicated that the minor allele of one of the variants in the haplotype (rs17713054) protects against HIV infection. We thus expected this allele to be over-represented in highly exposed individuals who remain uninfected (exposed seronegative individuals, ESN). To perform a meta-analysis, we genotyped rs17713054 in three ESN cohorts of European ancestry exposed to HIV through different routes. No evidence of association was detected in the single cohorts. The meta-analysis also failed to detect any effect of the variant on protection from HIV-1. The same results were obtained in a Cox-regression analysis for the time to seroconversion. An in-vitro infection assay did not detect differences in viral replication as a function of rs17713054 genotype status. We conclude that the rs17713054 minor allele is not associated with the ESN phenotype and does not modulate HIV infection in vitro.

Significance Statement The susceptibility to and severity of infectious diseases can be modulated by host genetic factors. In turn, the selective pressure exerted by pathogens in the past, as well as admixture with Neanderthals, contributed to the genetic makeup of human populations. An extended haplotype on chromosome 3 was inherited from Neanderthals and is a major risk factor for severe COVID-19. Recent data indicated that the same haplotype confers protection against HIV infection. We performed genetic association analyses of highly exposed HIV-negative individuals, as well as in vitro infection assays. Our data do not support a role of the Neanderthal haplotype in HIV infection susceptibility. Further data, in diverse ethnic backgrounds, will be necessary to clarify whether the chromosome 3 haplotype modulates the susceptibility (or severity) to HIV or other infectious agents.

## Introduction

The susceptibility to and the severity of many infectious diseases are modulated by host genetics. This is also true for pandemic pathogens such as SARS-CoV-2 and HIV-1. The strongest genetic risk factor for severe COVID-19 is a haplotype of Neanderthal-derived alleles on chromosome 3 (1, 2). Variants in the haplotype are in tight linkage disequilibrium and confer an odds ratio (OR) for requiring hospitalization after SARS-COV-2 infection of 1.6. The risk haplotype encompasses a cluster of genes encoding chemokine receptors. Recently, a study based on a meta-analysis of three large biobanks indicated that the minor allele (A) of a variant (rs17713054) within the risk haplotype confers a 27% decreased risk of HIV infection, which equates to an OR of 0.73 (3). The study also showed that the minor allele of this variant reduces the expression of several chemokine receptors, including CCR5, which functions as a co-receptor for HIV-1 (3). Indeed, homozygosity for a 32 bp deletion in the *CCR5* gene (*CCR5Δ32*) has been reliably associated with protection from HIV-1 infection (4). However, *CCR5Δ32* homozygous individuals are rare in all human populations and additional genetic factors are thought to explain why a proportion of highly exposed individuals does not seroconvert (exposed seronegative individuals, ESN) (5). Nonetheless, genome-wide association studies (GWASs) have failed to identify other genetic modulators of HIV acquisition (4), which is generally interpreted as evidence that the effects associated with common variants are of low penetrance (4).

## Results and discussion

We set out to confirm the association between rs17713054 and HIV-1 infection in three previously enrolled cohorts of ESN individuals. Such cohorts include subjects of European ancestry exposed to HIV by distinct routes. The first cohort comprises 173 Spanish injection drug users (IDUs). All of them are HCV-positive, but 85 remained HIV-1 negative despite multiple exposures through needle sharing. The two other cohorts include subjects exposed via sexual routes: 118 heterosexual Italian subjects who have a history of unprotected sex with their seropositive partners (to be compared to 343 Italian healthy controls, HCs) and 426 men who have sex with men (MSM) from the United States, 246 of whom remained seronegative after 36 months of follow-up.

Whereas the power to detect an association in the individual cohorts is expected to be low, we calculated the power for a meta-analysis considering an additive effect and an OR of 0.73 [as in (3)]. The three cohorts have an average of 149 ESN and 203 HIV+ or HC subjects. For this sample sizes, the power of a fixed-effects meta-analysis was equal to 79.33%. The power of a random-effects meta-analysis with low heterogeneity was 67.32%. These estimates may be considered conservative, as the OR was derived from general population datasets, whereas we have enrolled individuals with a clear resistance phenotype.

We thus genotyped rs17713054 in the three cohorts and, in line with previous data, we found that the minor allele frequency (MAF) ranged from ∼0.06 to ∼0.09 (2, 3). Using a logistic regression model, we observed no association with HIV-1 infection (Table 1). The results of the association analyses were combined through a random effect meta-analysis, which revealed no heterogeneity among samples (Cochrane’s Q *P*-value = 0.92, I2 = 0) and yielded an association *P*-value of 0.34 with an OR of 0.85 [95% CI: 0.61–1.18] (Table 1).

**Table 1.** Association of rs17713054 with HIV-1 infection susceptibility in three cohorts.

**Sample**	**Genotype counts (AA/AG/GG)**		**MAF (A allele)**		**OR (95% CI)** *^a^*	***P*** *^b^*	**Meta-analysis *P* and OR (95% CI)** *^c^*
IDU (Spain)	**ESN**	**HIV**	**ESN**	**HIV**	0.96 (0.44–2.09)	0.92	0.34, 0.85 (0.61–1.18)
	0/14/71	1/12/75	0.082	0.080			
SexExp (Italy)	**ESN**	**HC**	**ESN**	**HC**	0.86 (0.53–1.41)	0.56	
	3/17/98	3/52/288	0.097	0.085			
MSM (USA)	**ESN**	**HIV**	**ESN**	**HIV**	0.79 (0.46–1.35)	0.39	
	1/37/208	1/21/158	0.079	0.064			

a OR for an additive model with 95% CIs.

b Logistic regression *P*-values (additive model).

c Random-effect meta-analysis *P*-value and OR.

**Fig. 1.**
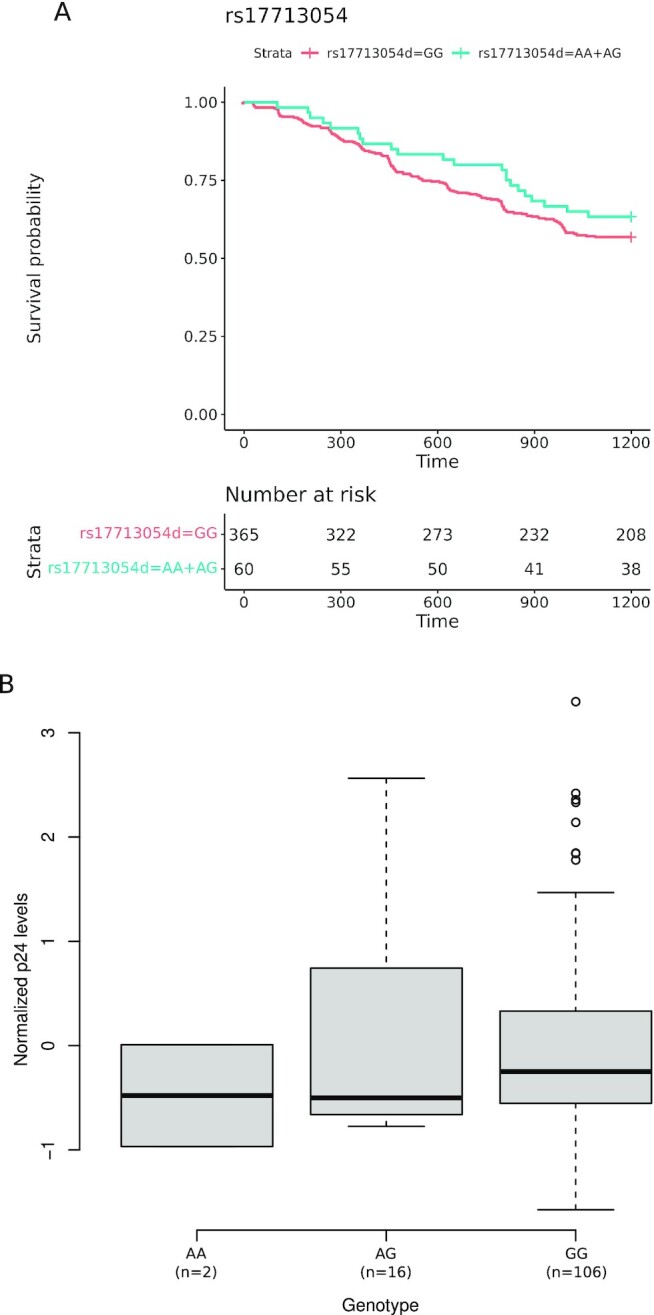
Cox regression and p24 levels. (A) Kaplan–Meier plot of rate of HIV-1 seroconversion according to rs17713054 genotypes. Survival curves were compared under a dominant model for (AA + AG versus GG) by the log-rank test (*P*-value = 0.29). (B) Box-and-whisker plot of p24 antigen level data are shown as a function of rs17713054 genotype in standard box-and-whisker plot representation (thick line: median; box: quartiles; whiskers: 1.5 × interquartile range).

For the MSM cohort, the time to seroconversion was also available. The seroconversion probabilities were calculated using the Kaplan–Meier method and a Cox regression model, which revealed a nonsignificant association of the Single nucleotide polymorphism (SNP) with the rate of HIV infection during the follow-up (hazard OR = 0.79 [95% CI: 0.50–1.22], long rank *P*-value = 0.30) (Fig. 1A).

Finally, to verify whether rs17713054 affects HIV-1 replication in vitro, we performed infection assays. Specifically, Peripheral blood mononuclear cells (PBMCs) from 124 Italian HC subjects were cultured and infected with HIV-1Ba-L. Viral replication was assessed after 3 days by measuring viral p24 levels produced by the infected cells. Results indicated that the A allele is not associated with significantly lower p24 antigen levels (One-way ANOVA, *F* = 0.32, *P*-value = 0.72) (Fig. 1B).

Overall, our data provide no evidence that rs17713054 affords protection from HIV infection. Although our study was not powered to detect associations in the single cohorts, the sample sizes for the meta-analysis was estimated to be adequate for an OR of 0.73, especially in the case of no heterogeneity, as expected for three samples of European ancestry. Moreover, in-vitro infection assays revealed no difference in viral replication in subjects with different rs17713054 genotypes. Our data are also in agreement with a large-scale GWAS study for HIV acquisition that analyzed ∼8 million variants and detected no signals on chromosome 3 (6).

It was previously shown that the minor A allele of rs17713054 is part of an extended haplotype that was inherited from Neanderthals. To date, this haplotype represents the major genetic risk factor associated with severe symptoms after SARS-CoV-2 infection. Differences in the frequency of the risk haplotype in South and East Asia led to the suggestion that some selective pressure, possibly related to cholera, drove its spread in South Asians. If this is the case, we expect the haplotype to be associated with some selective advantage. As noted elsewhere, HIV cannot be regarded as exerting selective pressure, as its introduction to human populations is too recent. Moreover, data herein do not support an effect of this haplotype, or at least of rs17713054, on HIV acquisition. Additional analyses will be required to determine whether these Neanderthal alleles confer protection against other infectious agents.

## Materials and methods

IDUs were enrolled in prospective cohort studies in Spain (Valme Hospital, Sevilla), as previously described (7, 8). They were all males and had shared needles for >3 months. Concurrent markers of hepatitis C virus (HCV) infection were present in 100% of IDU subjects. For Italian ESN, inclusion criteria were a history of multiple unprotected sexual episodes for more than 4 years at the time of the enrolment, with at least three episodes of at-risk intercourse within 4 months prior to study entry and an average of 30 (range, 18 to >100) reported unprotected sexual contacts per year (9). ESN subjects and HCs were recruited at the S. M. Annunziata Hospital, Florence; all of them were Italian of European origin. Finally, this study includes MSM of European ancestry from the United States. They were volunteers participating in a placebo-controlled phase 3 trial of a prophylactic vaccine against HIV-1 infection (clinical trial identifier: NCT00002441). The gp120 HIV-1 envelope-based candidate vaccine was based on two recombinant proteins (MN and GNE8). Individuals were eligible for entering the trial if they had anal intercourse during the preceding 12 months and did not have a monogamous sexual relationship with an HIV-1 seronegative partner. Additionally, intravenous drug use was a criterion for exclusion (10, 11). The infection rates were 6.7% in vaccinees versus 7.0% in placebo recipients; vaccine efficacy was considered proximal to zero. Subsequent follow-up revealed no differences between treatment arms in secondary endpoints including viral loads, time to antiretroviral therapy or HIV-1 strains. In this study, we included a random sample of volunteers with DNA samples available that were followed for up to 1,200 days after vaccine or placebo administration (10).

This study was approved by the Institutional Review Board of the Province of Jaen, Junta de Andalucia (GEN-VIH/0646-N-20 version 1 of 2020 March 9 and Protocol “Identificación de factores genéticos de Resistencia innata a la infección por VIH-1” of 2018 July 26) and was designed and performed according to the principles of the Helsinki Declaration.

All patients and healthy blood donors provided written informed consent to participate in this study.

Details on genotyping, statistical analyses, and in-vitro infection assays are available in the Extended Methods and data.

## Supplementary Material

pgac138_Supplemental_File

## Data Availability

The study data are included in the article (Table 1) and/or in the Extended Methods and data (p24 data).
