# Supplementary material for: No association of a risk variant for severe COVID-19 with HIV protection in three cohorts of highly exposed individuals
Source: PNAS Nexus. 2022 Aug 4;1(3):pgac138. doi: 10.1093/pnasnexus/pgac138 (PMC9896871; doi:10.1093/pnasnexus/pgac138)
Supplement: pgac138_Supplemental_File [file pgac138_supplemental_file.docx]

**Extended Methods and data for “No association of a risk variant for severe COVID-19 with HIV protection in three cohorts of highly-exposed individuals”**

**Genotyping and statistical analysis**

Genomic DNA was used as the template for PCR amplification using TaqMan probes specifically designed to perform a SNP genotyping assay for rs17713054 (G/A) (TaqMan® Assay:  C_3128636_20) and using the allelic discrimination real-time PCR method. Assays were performed using a QuantStudio™ 3 Real-Time PCR System, Applied Biosystems by ThermoFisher Scientific. The variant complied to Hardy-Weinberg equilibrium in all samples.

The power for the meta-analysis was calculated using the ‘power.analysis’ function in the **dmetar R package (10.5281/zenodo.2551803, 10.1002/9780470743386).**

Genetic association analyses were performed by logistic regression and results from the three cohorts were combined using a random-effect meta-analysis; these analyses were performed using PLINK (1).

**HIV infection assay**

PBMCs from 124 HC subjects were separated on a lymphocyte separation medium; 10×10^6^ cells/mL were cultured for 2 days at 37°C and 5% CO_2_ in RPMI 1640 containing FBS (20%), phytohemagglutinin, and interleukin (IL)—2. After viability assessment, 2.5×10^6^ cells were resuspended in a medium containing 1 ng of HIV-1_Ba-L_ p24 viral input/10^6^ PBMC and incubated for 3 h at 37°C. Cells were then washed and resuspended in 3 mL of complete medium with IL-2. Cells were plated in 24-well tissue culture plates and incubated at 37°C and 5% CO_2_. After 3 days, absolute levels of p24 were measured in supernatants using the Alliance HIV-1 p24 ELISA Kit. PBMCs from the 124 subjects were infected in five independent experiments. To account for minor differences in virus titre, p24 levels were normalized within experiments. HIV-1_Ba-L_ was provided through the EU programme EVA Centre for AIDS Reagents NIBSC, UK.

**References**

1. Purcell S*, et al* (2007) PLINK: A tool set for whole-genome association and population-based linkage analyses*. Am J Hum Genet* 81(3): 559-575.

**Supplementary data- p24 levels and rs17713054 genotype in 124 healthy controls**

| **Normalized p24 level** | rs17713054 genotype |
| --- | --- |
| -0.4326 | GG |
| -0.4211 | GG |
| -0.1201 | GG |
| 2.3577 | GG |
| 0.3667 | GG |
| -0.7740 | AG |
| -0.6793 | GG |
| -1.1243 | GG |
| 0.7751 | GG |
| 0.0518 | GG |
| -0.9260 | GG |
| 0.6632 | GG |
| 0.0094 | AA |
| -0.8871 | GG |
| -0.4793 | GG |
| 0.1615 | GG |
| 0.9674 | GG |
| -1.5733 | GG |
| 0.2845 | GG |
| 1.7798 | GG |
| 0.1008 | GG |
| 0.0242 | GG |
| -1.5654 | GG |
| -1.1114 | GG |
| 0.6472 | GG |
| 1.8421 | GG |
| -1.0301 | GG |
| 0.2167 | GG |
| 0.4307 | GG |
| 2.3318 | GG |
| -0.1853 | GG |
| -0.7551 | GG |
| 0.6500 | GG |
| -0.4426 | GG |
| 0.2808 | GG |
| -1.3037 | GG |
| 0.1253 | GG |
| 1.8470 | GG |
| -0.1418 | GG |
| -0.7000 | AG |
| -0.5752 | GG |
| -0.7123 | GG |
| 0.9158 | GG |
| -0.6335 | GG |
| -0.3639 | GG |
| -0.4599 | GG |
| -0.3628 | GG |
| 0.0016 | GG |
| -0.5146 | GG |
| 2.4194 | GG |
| -0.7419 | GG |
| 1.3674 | GG |
| -0.6930 | AG |
| -0.4004 | GG |
| 1.1904 | AG |
| 0.0152 | AG |
| -0.4626 | GG |
| 1.7217 | AG |
| -0.7064 | AG |
| -0.5679 | GG |
| 0.3009 | GG |
| -0.7301 | GG |
| 0.3326 | GG |
| 0.3090 | GG |
| -0.0939 | GG |
| -0.8815 | GG |
| 1.4675 | GG |
| -0.4813 | GG |
| -0.3336 | GG |
| 0.2241 | GG |
| -0.5291 | GG |
| 0.7849 | GG |
| -0.0123 | GG |
| -0.7605 | GG |
| -0.1760 | GG |
| 0.1762 | GG |
| -0.5317 | GG |
| -0.4883 | GG |
| -0.2447 | GG |
| 0.4276 | GG |
| -0.5453 | GG |
| -0.0423 | GG |
| 0.0541 | GG |
| 1.3313 | AG |
| 0.3227 | GG |
| -0.1551 | GG |
| -0.4103 | GG |
| -0.2536 | GG |
| 0.9502 | GG |
| -0.4038 | GG |
| -0.0428 | GG |
| -0.4970 | GG |
| -0.5570 | GG |
| -0.5520 | GG |
| -0.5716 | AG |
| 0.4548 | GG |
| -0.4950 | AG |
| -0.5334 | AG |
| 0.8847 | GG |
| -0.5838 | GG |
| -0.5071 | AG |
| -0.2797 | GG |
| -0.6651 | GG |
| -0.6452 | GG |
| -0.6659 | GG |
| 2.5644 | AG |
| -0.4697 | GG |
| -0.3513 | AG |
| -0.3440 | GG |
| 0.2958 | AG |
| -0.6408 | GG |
| -0.5682 | GG |
| 1.2978 | GG |
| -0.2410 | GG |
| 0.8186 | GG |
| 3.2980 | GG |
| -0.6005 | GG |
| -0.4408 | GG |
| -0.2766 | GG |
| -0.4285 | GG |
| -0.6272 | AG |
| 1.1991 | GG |
| -0.9663 | AA |
| 2.1409 | GG |
